# Computer-aided detection of arrhythmogenic sites in post-ischemic ventricular tachycardia

**DOI:** 10.1038/s41598-023-33866-w

**Published:** 2023-04-27

**Authors:** Giulia Baldazzi, Marco Orrù, Graziana Viola, Danilo Pani

**Affiliations:** 1 Medical Devices and Signal Processing (MeDSP) Lab, Department of Electrical and Electronic Engineering (DIEE), University of Cagliari, Cagliari, Italy; 2 Department of Informatics, Bioengineering, Robotics and Systems Engineering (DIBRIS), University of Genoa, Genoa, Italy; 3 Department of Cardiology, Santissima Annunziata Hospital, Sassari, Italy

**Keywords:** Biomedical engineering, Ventricular tachycardia

## Abstract

Nowadays, catheter-based ablation in patients with post-ischemic ventricular tachycardia (VT) is performed in arrhythmogenic sites identified by electrophysiologists by visual inspection during electroanatomic mapping. This work aims to present the development of machine learning tools aiming at supporting clinicians in the identification of arrhythmogenic sites by exploiting innovative features that belong to different domains. This study included 1584 bipolar electrograms from nine patients affected by post-ischemic VT. Different features were extracted in the time, time scale, frequency, and spatial domains and used to train different supervised classifiers. Classification results showed high performance, revealing robustness across the different classifiers in terms of accuracy, true positive, and false positive rates. The combination of multi-domain features with the ensemble tree is the most effective solution, exhibiting accuracies above 93% in the 10-time 10-fold cross-validation and 84% in the leave-one-subject-out validation. Results confirmed the effectiveness of the proposed features and their potential use in a computer-aided system for the detection of arrhythmogenic sites. This work demonstrates for the first time the usefulness of supervised machine learning for the detection of arrhythmogenic sites in post-ischemic VT patients, thus enabling the development of computer-aided systems to reduce operator dependence and errors, thereby possibly improving clinical outcomes.

## Introduction

Ventricular tachycardia (VT) is a ventricular arrhythmia caused by structural ischemic or non-ischemic heart diseases, which is associated with an increased risk of sudden death^1–3^. The most common cause of VT is a previous ischemic event that induces an irreversible scar in the ventricle, thus significantly altering the ventricular myocardial electrical function and reshaping its propagation pathways^4^. Different approaches to treat VTs can be used according to the patient’s conditions^5^. Among the various possible therapeutic treatments^5–8^, trans-catheter ablation is an effective option for different forms of VT^9–12^, particularly for the scar-related ones^8,13–15^. However, it frequently requires more than one procedure to be completed^7,12,16,17^and the recurrence rate after clinical procedures still ranges between 30% up to more than 40%^18–20^. Generally, trans-catheter ablation inhibits VT onset by electrically silencing myocardial sites that trigger or sustain the arrhythmia^21–23^ by means of radiofrequency energy, which produces electrically inactive thermal lesions, i.e., scars. To carry out the ablation procedures, various strategies to spatially identify the target points have been outlined, including several types of electroanatomic (EA) maps^24^. Substrate-guided mapping allows fast identification of the targets under hemodynamically stable conditions^13–15^. It is based on the identification of arrhythmogenic sites in low-voltage, slow conduction areas during sinus rhythm^16^. In this approach, the target points are mainly identified as abnormal electrical conduction pathways that lead to the so-called abnormal ventricular potentials (AVPs) in the electrograms (EGMs)^13,25–28^. Nowadays, AVPs are visually identified by clinicians during electrophysiological studies and manually tagged on the EA maps to perform the ablation. This approach requires the operator to examine each acquired signal and recognize AVPs among a large number of recorded EGMs. On the basis of this premise, electrophysiological procedures result in a complicated and significantly time-consuming process, and their outcomes are highly dependent on the operators’ expertise.

Several supporting mapping tools have been proposed to mitigate these problems, such as the CARTO 3 ripple mapping^29^, the Rhythmia HDx mapping and the Ensite Precision automatic mapping^30^, the Ensite Precision fractionation mapping algorithm^31^, and the Rhythmia Lumipoint algorithm^32^. The literature presents a wide range of studies that investigate the analysis of intracardiac EGM data to support clinicians in the identification of ablation targets. In this regard, existing literature demonstrated the effectiveness of using parameters extracted from EGMs to define critical areas related to VT, including voltage, fragmentation and duration criteria^33^, the simultaneous amplitude frequency EGM transformation (SAFE-T) mapping^34^, the fragmentation map^35^, the combination of voltage limit adjustment with fast Fourier transform^36^, the automated fractionation detection algorithm^37,38^, the re-entry vulnerability index^39,40^ and the recent algorithm for 3D visualization of EGM duration^41^. These studies clearly indicated the possibility of defining cut-off ranges for highlighting VT critical sites, with sensitivities and accuracies ranging from 65 to 100% in detecting ablation areas^33,41^, fractionated signals^38^, VT sites of origin^39,40^, or VT isthmuses^36^, or proving statistically significant differences between control groups and VT patients^34,35,37^. Moreover, many alternative targeting strategies have also been presented in clinical prospective studies, such as the decrement evoked potential mapping^42–44^, the high-density mapping based on deceleration zones^45^, and the hidden slow conduction analysis^46,47^. In these works, the application of alternative targeting strategies for ablation obtained sensitivities between 50 and 61% in detecting areas initiating and maintaining VT^42,43^, with more than 75% of survival rate^45,47^, thus demonstrating their effectiveness. Although several new methods have been developed to identify and map VT arrhythmogenic areas aiming at improving the efficiency of arrhythmogenic substrate identification and, possibly, the clinical outcome, no artificial intelligence (AI) approaches based on multi-domain feature have been investigated so far, except the preliminarily investigations reported in^48,49^.

This work aims to explore the adoption of AI tools for the identification of arrhythmogenic sites in VT mapping procedures, based on the study of conventional and unconventional features belonging to time, time scale, frequency, and spatial domains. Well-trained AI-based clinical decision support systems are expressly conceived to analyze huge amounts of data to support decision-making. In the context of the proposed research, the adoption of AI tools, capable of generalizability, can allow highlighting EGM patterns that could escape to the trained eye by rapid visual inspection, as asked to electrophysiologists during VT mapping procedures. The assessment of the effectiveness of the proposed features and the validation of the overall approach were performed by considering three supervised classification methods for discriminating between AVPs and physiological potentials.

## Materials and methods

### Dataset

A dataset collected from nine patients (78% male, age: 66 ± 10 years old, ejection fraction: 29.4% ± 5.8%) with post-ischemic VT between 2017 and 2018 at the San Francesco Hospital (Nuoro, Italy) was created. A retrospective study on anonymized patient signals was approved by the Independent Ethical Committee of the Azienda Tutela Salute, Sardegna (Prot. n. 351/2021/CE, date of approval: 13/07/2021) and performed following the principles outlined in the 1975 Helsinki Declaration, as revised in 2000. All the participants provided their signed informed consent.

The CARTO 3 system (Biosense Webster, Inc., Diamond Bar, California) was used to perform the recordings in sinus rhythm, during left ventricle (LV) EA mapping. Subsequently, radiofrequency ablation was performed according to the current guidelines. Bipolar EGMs were recorded using PentaRay (Biosense Webster, Inc., Diamond Bar, California) 2-6-2 mm, ThermoCool SmartTouch and ThermoCool SmartTouch SF (Biosense Webster, Inc., Diamond Bar, California). The CARTO 3 system sampled all signals at 1 kHz and performed band-pass filtering between 16 and 500 Hz. Even though the length of each exported recording was 2.5 s, for each EGM, a segment around the last cardiac cycle identified by the reference annotation (i.e., a fiducial point in the cardiac cycle defined according to the ventricular activity) was considered to include only acquisitions performed when the catheter was in contact with the endocardium. All EGM segments were manually annotated as physiological or AVPs by an experienced electrophysiologist blinded to the case and to the position of the potentials in the EA map with the use of an ad-hoc MATLAB graphical user interface that was implemented for this purpose in previous studies^49,50^. It should be also pointed out that all EGMs without pathological deflections (i.e., exhibiting a “normal” behaviour) belonging not only to healthy tissue (i.e., from undamaged ventricular areas), but also to border-zone and scar substrates, were annotated as physiological.

We chose to perform a blind annotation to the EGM localization on the EA map, so that the clinician may recognize the different potentials without any influence from their spatial localization, but solely looking at the amplitude and morphology of the examined EGM. In fact, the final objective of the annotation process was the identification of EGMs with normal and abnormal behaviors, being the AVPs correlated with arrhythmogenic sites. Surely, the spatial localization must be taken into account when the ablation targets should be identified, which however is one step further, and beyond the scope of the present work. The dataset consisted of 1584 EGMs, including only exported signals that were spatially projected in the reconstructed LV EA map with a projection distance below 8 mm, for a correct localization of the EGM on the EA map. This threshold was selected by considering the recommendations provided by the CARTO 3 system technical documentation^51^. There, 8 mm is the threshold adopted to graphically warn, by means of blue markers, the operator about EA points with less accurate spatial localization. Clearly, the lower the projection distance, the more reliable is the EA point anatomical localization. Among all EGMs, 618 (i.e., 39%) AVPs were identified, along with 966 (i.e., 61%) physiological potentials. As the VT arrhythmogenic areas are typically restricted when compared with healthy myocardial tissue, the dataset composition was intrinsically unbalanced toward the physiological signal class.

**Figure 1:**
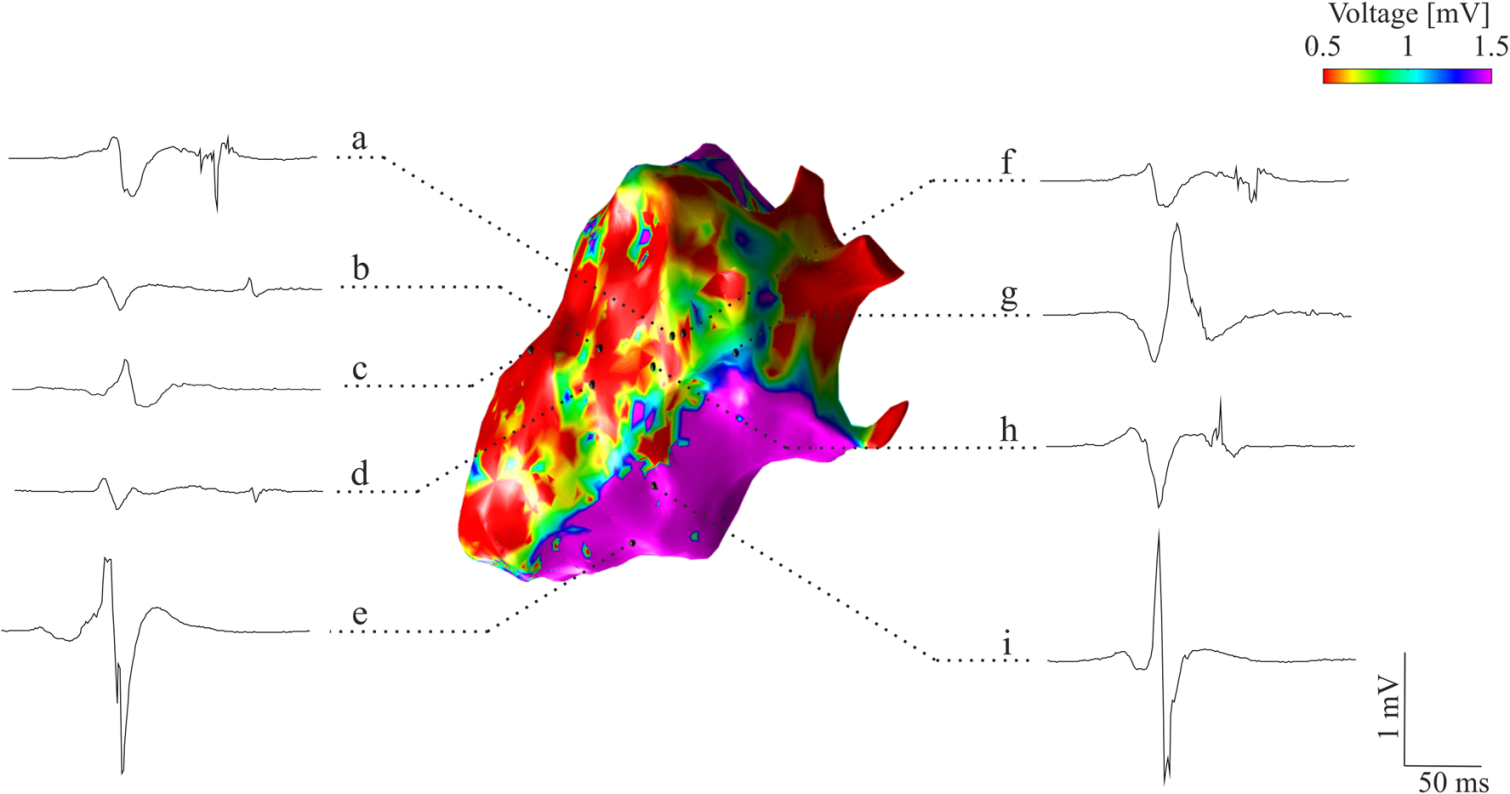
Some examples of EGMs included in the dataset, with their spatial localization on the EA voltage map. Specifically, AVPs with deflections during and after the QRS (**a**,**f**), spreading after the end of the QRS depolarization (**b**,**d**), and completely falling within the surface QRS (**h**) are presented along with physiological potentials from scar (**c**), border zone (**g**), and healthy tissue (**e**,**i**). The EA voltage map was generated from CARTO 3 export files in MATLAB v2022a (MathWorks Inc., MA, USA).

Specifically, different types of potentials fell into the AVP class, mainly enclosing endocardial bipolar deflections spreading after the end of the corresponding surface QRS depolarization, those starting during the corresponding surface QRS depolarization but vanishing after its end, and early EGM deflections completely falling within the corresponding surface QRS depolarization boundaries^50,52^. Some examples of EGMs included in the adopted dataset are shown in Fig. 1 with their relative spatial location on the voltage EA map.

### Feature extraction

In light of our prior investigations^48–50^, multiple quantitative features that characterize the EGM were extracted and used to train supervised classification models to distinguish pathological from physiological potentials. Four types of features were identified, which were obtained from the time, frequency, time-scale, and spatial domains, as detailed hereinafter.

#### Features from time domain

In the time domain, two features were extracted for each bipolar EGM segment: the peak-to-peak amplitude (A_pp_) and a fragmentation measure. The A_pp_ has been largely exploited in the scientific literature^7,14,53,54^ and as such, it was introduced to enable the differentiation between high voltage EGMs (A_pp_ > 1.5 mV), which are typically associated with healthy myocardium, and signals originating from scar and border zone regions, which are generally characterized by lower voltages (i.e., A_pp_ < 0.5 mV and 1.5 mV < A_pp_ < 0.5 mV, respectively)^53^. To determine the A_pp_, we analyzed each EGM within a 350-ms window around the reference annotation (i.e., 50 ms before and 300 ms after), and computed the difference between the largest EGM positive deflection (A_p_^+^) and the absolute value of the largest negative deflection (A_p_^−^) from the isoelectric within that window, as:

1 $$A_{pp} = \left( {A_{p}^{ + } } \right) - \left( {A_{p}^{ - } } \right)$$

The fragmentation measure was adopted because fractionated potentials indicate slow and inhomogeneous conduction of the electrical activation locally^55,56^. Even though it has not been analytically described, this measure has been frequently used in the literature^22,31,33,35,57,58^. In this study, fragmentation was defined as the total number of peaks in the 350-ms long EGM segment, as in^48,49^. To do this, the 350-ms EGM segment was rectified by extracting its absolute value, and all the peaks exceeding a given amplitude threshold were counted. In this computation, the threshold T was identified as 75% of the mean absolute deviation (mad) of the rectified EGM, as:

2 $$T = 0.75* mad\left( {\left| {EGM} \right|} \right)$$

#### Features from time-scale domain

Two features have been also computed in the time-scale domain using continuous wavelet transform (CWT), which guarantees a good resolution both in time and in frequency domains. At first, a preliminary undecimated wavelet denoising^48^ was performed, by considering a 2-level decomposition, the Daubechies-2 mother wavelet, the Universal threshold ($\theta_{j}$) and the soft thresholding of the detail coefficients (cD), as:

3 $$\theta_{j} = \sigma_{j} \cdot \sqrt {2\ln \left( N \right)}$$

4 $$\overline{{cD_{j,k} }} = \left\{ {\begin{array}{*{20}l} {sign\left( {cD_{j,k} } \right)\left( {\left| {cD_{j,k} } \right| - \theta_{j} } \right)} \hfill & { if \left| {cD_{j,k} } \right| \ge \theta_{j} } \hfill \\ 0 \hfill & {otherwise} \hfill \\ \end{array} } \right.$$

where cD_j,k_ indicates the k-th detail coefficient at level j, while σ_j_ was defined as:

5 $$\sigma_{j} = 1.4826 \cdot median\left( {\left| {cD_{j} } \right|} \right)$$

Undecimated wavelet transform was used to achieve translation invariance, as the morphological analysis is affected by the loss of this property in conventional decimated schemes. This denoising stage, which is applied to all the spectral contents from about 125–500 Hz (according to the chosen level of decomposition), was aimed at reducing small noisy fluctuations of the EGMs before computing the features in the time-scale domain. Then, the CWT was performed with Daubechies-2 mother wavelet and scales from 2 to 35, and the sum and the standard deviation of the average powers associated with the five most powerful scales were computed across the scales of the CWT decomposition and considered as features. Remarkably, although on the overall dataset the five most powerful scales range from 31 to 35 (which respectively correspond to about 22.2, 21.5, 20.8, 20.2 and 19.6 Hz), the identification of the five most powerful scales was performed independently for each EGM. As such, those scales vary from EGM to EGM independently, according to their spectral content.

#### Features from frequency domain

On the basis of recent evidence^50^, several spectral signatures of EGMs were considered, including the relative power in several frequency sub-bands and other metrics relying on the morphology of the EGM spectrum.

Specifically, after the power spectral density (PSD) computation, the relative power content of each EGM was considered, according to a sub-band analysis approach with a 20 Hz granularity. Specifically, in each sub-band, the relative power was determined as the percentage ratio between the absolute power contained in that sub-band and the overall power content up to 320 Hz, according to a previous study^50^. Indeed, relative powers allowed eliminating the influence of the signal amplitude, which conversely is already accounted for in the time domain features. Relative power showed to be useful in distinguishing between AVPs and physiological potentials^59^, and to detect statistically significant differences among them in several frequency ranges^50^. Accordingly, the relative powers in the 20 Hz sub-bands between 0 and 320 Hz were considered as features. However, in this analysis, the sub-band 20–40 Hz was not included, as no statistical difference between physiological signals and AVPs was found for that sub-band so far^50^.

Furthermore, some interesting statistical differences between physiological potentials and AVPs emerged in the literature^50^ also by looking at the PSD morphologies through some synthetic metrics, as the mean frequency (MNF), the peak frequency (PKF), the mean spectral power (MNP), and the power spectrum ratio (PSR). Specifically, the MNF expresses if the power spectral contents are mostly localized at higher or lower frequencies, the PKF identifies the frequency in which the PSD maximum occurs, and the MNP and the PSR quantify the average power below 320 Hz and in the range around the PKF, respectively^50,60^. As such, in this study, we included all these metrics as features in the frequency domain.

#### Features from spatial domain

Different features related to the spatial localization of each EGM were deduced from the EA maps to provide more insights on the electrical characteristics of the surrounding endocardial area. Arrhythmogenic sites are often located in altered conduction areas, such as border zones or dense scars, rather than randomly distributed all over the EA map. Therefore, spatial features were added to fill the information gap caused by the exclusive adoption of signal morphology based features.

Specifically, the spatial information was retrieved from two perspectives: the voltage map (substrate map) and the local activation time and signal conduction properties (LAT map).

**Figure 2:**
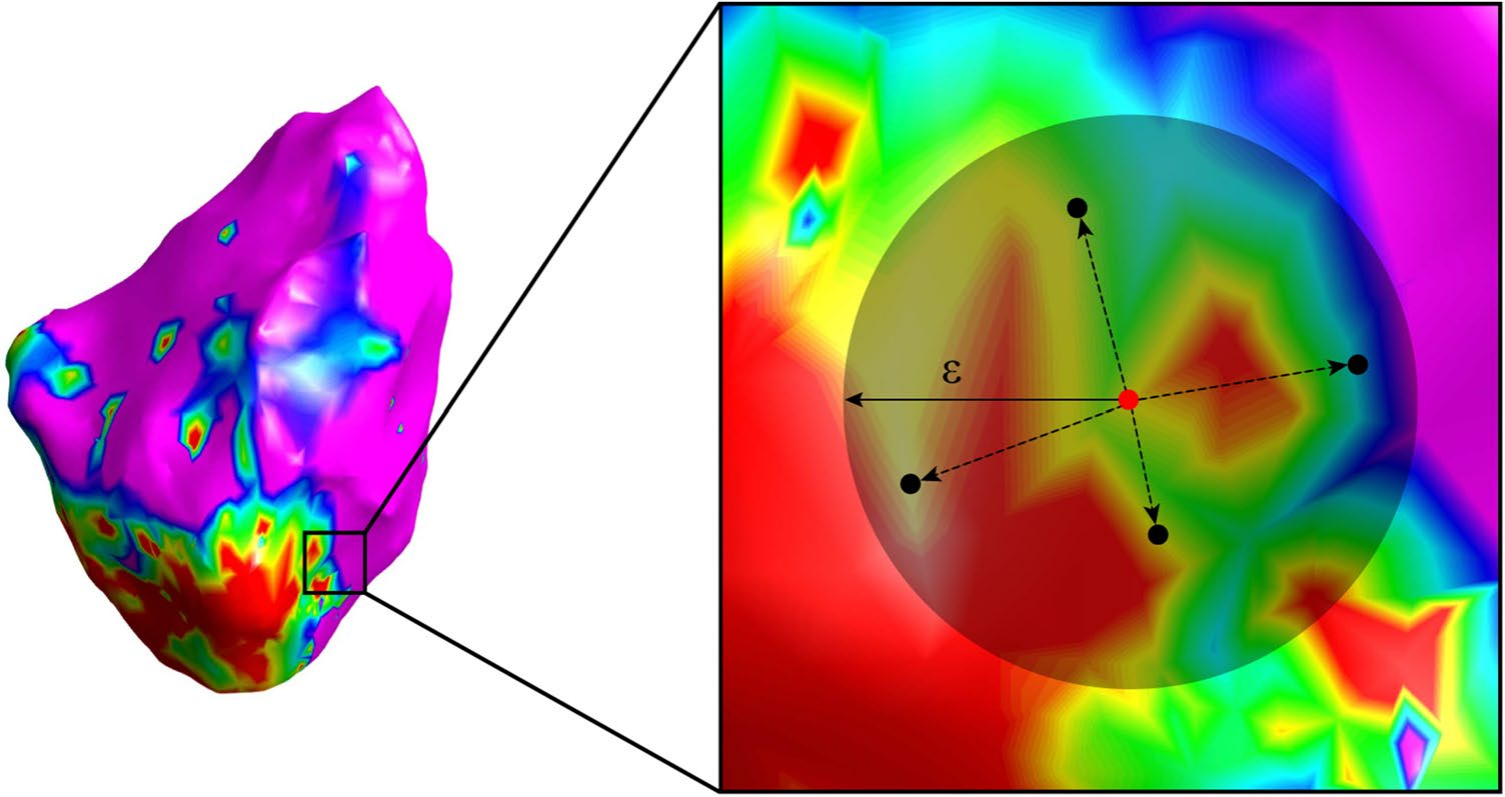
Schematic representation of the 6-mm circular area *C*_*P*_ determining the neighbors of the point P (in red) into the EA voltage map. As can be seen, the neighborhood is centered on the location of the EGM of interest (P) and spans for a radius equal to $\epsilon$  = 6 mm. Black dotted arrows represent the distances between each neighbor point and P. All EGM points falling within the selected area are exploited for the computation of the spatial features of P. The EA voltage map was created from CARTO 3 export files in MATLAB v2022a (MathWorks Inc., MA, USA).

First, we considered the circular area *C*_*P*_ on the EA map, with a radius $\epsilon$ and centered in P, where P is the projection on the map of the considered EGM acquisition point (see Fig. 2). According to preliminary investigations (data not shown), $\epsilon$ was set to 6 mm. For each EGM, all the neighbors (other acquisition points for which an EGM is available that fall within this *C*_*P*_ area) were considered to extract the following features. An average number of 55 neighbors (95% CI, 51–58) was identified in each *C*_*P*_*.* On the substrate map, we extracted:

- The mean bipolar voltage, computed as the mean value of the peak-to-peak amplitude A_pp_ exhibited by all EGMs in *C*_*P*_;
- The weighted mean bipolar voltage, evaluated as before but weighting the neighbors’ contribution by the exponential function $e^{ - x}$, where x represents the Euclidean distance of the different points in *C*_*P*_ from the point P;
- The standard deviation of the A_pp_ exhibited by the bipolar EGMs acquired in *C*_*P*_;
- The number of neighbors associated with scar, border zone, and healthy tissues. This number was normalized with respect to the total neighbors identified in *C*_*P*_, to provide features independent from the density of the points acquired during the EA mapping procedure.

On the LAT map, we extracted three additional spatial features for each EGM:

- The LAT value, i.e., the local activation time, as provided by the CARTO 3 exported files or, if not present, by considering the CARTO 3 interpolated LAT in the nearest EA points.
- The conduction velocity (CV), which was derived from the LAT information according to^61,62^, is intrinsically connected to electrophysiological characteristics of the myocardial substrate, especially in cases of arrhythmogenic causes^63^ and provides essential information on the speed and the direction of the wavefront propagation;
- The coherence of directions (CD) in *C*_*P*_, which was computed as the standard deviation of the angles between the CV vector of P and those of the neighbors. This feature was introduced to express the level of randomness in the direction of the wavefront conduction (the higher the CD value, and then the standard deviation of the abovementioned angles, the more disordered the conduction).

Finally, a standardization stage was performed on all features. As such, by considering each feature separately, and its values across the different EGMs (and patients) as a distribution, the standardization was performed according to the Z-score normalization method, by subtracting the mean and dividing by the standard deviation each value of such distribution. In this way, all the features belong to zero-mean, unit-variance distributions. This pre-processing step was adopted in order to get rid of any bias related to the different range of values that the input features may assume according to their definition, thus providing less sensitivity of the models to the scale of input features.

### Classification models

Three feature-based supervised classification models were trained for the recognition of AVPs and physiological potentials: support vector machine (SVM), K-nearest neighbors (KNN), and ensemble tree (ENS). First, a preliminary study for the choice of the SVM kernel and the number of neighbors for the KNN was conducted (see Supplementary Tables S1 and S2). On the basis of this study, the third-order polynomial kernel was chosen for the SVM, and 10 neighbors were considered for the KNN.

For all the three identified models, the MATLAB default parameters for the chosen classifiers were adopted. The rationale behind this choice is that parameters optimization on a limited-size dataset is prone to overfitting, and the goal of the research was mainly the assessment of the presented approach to solve the problem at stake rather than the achievement of the highest performance on the available dataset. Accordingly, we preferred a more robust classification model to the ideal classification performance by using optimization techniques or parameter tuning. Moreover, given the limited dataset size, the introduction of a validation set for parameter optimization would have further reduced test and training sets, affecting overall performance.

As regard such default parameters, for the SVM, the sequential minimal optimization routine along with a unitary penalty weight (i.e., box constraints) and no expected proportion of outliers in training data were used. For the KNN, a Euclidean neighbor searcher method with a maximum of 50 data points in the leaf node and no weighting method were imposed. For the ENS, the logit boost aggregation method with 100 ensemble learning cycles and 10 decision splits per tree (at maximum) were adopted.

### Feature selection

Feature selection was performed to understand if taking into account only the most relevant features may improve, worsen, or leave the classification results unchanged. The minimum redundancy maximum relevance (mRMR) feature selection method was adopted to highlight the most useful features in the set with respect to the response variable^64,65^, thus identifying the features that have highest correlation with the response but the lowest correlation among themselves. Feature selection was performed on the training set obtained in each fold and time for the 10-time 10-fold cross-validation scheme, leading to 100 assessments of the features. Then, a common set of features was retrieved as follows. Considering the relevance score provided by the mRMR algorithm for each feature, a score vector was generated and normalized between 0 and 1 at each iteration. Subsequently, a single score vector (*V*_*s*_) was obtained by summing the score for each feature, thus computing a unique relevance value. Then, *V*_*s*_ was sorted in descending order, and the contribution of each feature, from the most to the less important one, was cumulatively summed until 80% of the total relevance was reached. At this point, the first M features composing the 80% of the total relevance were considered as selected features for investigating the entire set both in the 10-time 10-fold and in the leave-one-subject-out cross-validation. The 80% threshold was imposed because it was found to be adequate for feature reduction according to preliminary investigations (data not shown).

### Performance evaluation

To provide an objective and quantitative evaluation of the proposed classification approaches, all classification methods were trained and tested in two ways.

First, a stratified 10-time 10-fold cross-validation scheme was used. This validation strategy was adopted to obtain an objective and robust evaluation of the performance by exploiting stratified partitions based on a common pool of instances coming from all the patients. It provides an unbiased result over a dataset characterized by a reduced number of instances. In this case, an equal number of instances of AVPs and physiological signals were randomly selected from the main pool to compose each fold, which accounts for 10% of the pool size. Then, nine folds were used for training and one for test, iteratively repeating training and test in order to cover all the permutations. Though this approach is conceived to reduce the bias of a single validation, a further unbiasing was pursued by repeating ten times this process. In this case, a different random selection of physiological EGMs (available in more instances than AVPs) to be inserted in the data pool was carried out, with the aim of performing an evaluation as generalizable as possible.

To provide an insight into the performance of our approach in a scenario that most resembles a real EA mapping application, a leave-one-subject-out cross-validation scheme was also explored. This approach allows for an accurate performance evaluation on EGMs from patients never seen by the classifier during the training phase, thus mimicking a real-world application in which the model would be exploited on patients never seen before, and for the evaluation of the impact due to eventual patient-specific characteristics. Each model was trained and tested nine times, each time using eight patients out of nine for training and the remaining one for testing. This latter analysis aimed to investigate if the models were possibly affected by patient-specific characteristics.

To perform an unbiased performance assessment, all models were trained and tested using the same sets of AVPs and physiological signals in each fold or patient-related partition of the dataset.

To evaluate the performance, five metrics were computed, i.e., the accuracy (ACC), the true positive rate (TPR) or sensitivity, the true negative rate (TNR) or specificity, the false positive rate (FPR), and the F1-score, as follows:

6 $$ACC{ } = \left( {TP{ } + { }TN} \right)/\left( {P{ } + { }N} \right)$$

7 $$TPR = TP/P$$

8 $$TNR = TN/N$$

9 $$FPR = FP/N$$

10 $$F1 - score{ } = { }2\left( {PPV \cdot TPR} \right)/\left( {PPV{ } + { }TPR} \right)$$

where $P$ and $N$ represent the total number of AVPs and physiological EGMs, respectively; $TP$ and $TN$ are the number of AVPs and physiological potentials that were correctly identified, respectively; $FP$ is the normal EGMs classified as AVPs; and $PPV$ is the positive predictive value or precision, defined as:

11 $$PPV{ } = { }TP/\left( {TP + FP} \right)$$

In the 10-time 10-fold cross-validation scheme the performance metrics were evaluated on the total number of $TP$, $TN$, $FP$, $P$, and $N$ obtained by summation over the different folds at each time. Therefore, all indexes were computed for each time, and results were reported as mean and standard deviation of the values obtained across the 10 iterations, thus yielding a better representation of the performance over the whole dataset.

Similarly, in the leave-one-subject-out case, summation was exploited over the different patient-related subsets, thus collecting a single value per metric. This choice was necessary to avoid computing percentages over strongly imbalanced subsets.

All computations were performed with MATLAB v2022a (MathWorks Inc., MA, USA).

## Results

**Figure 3:**
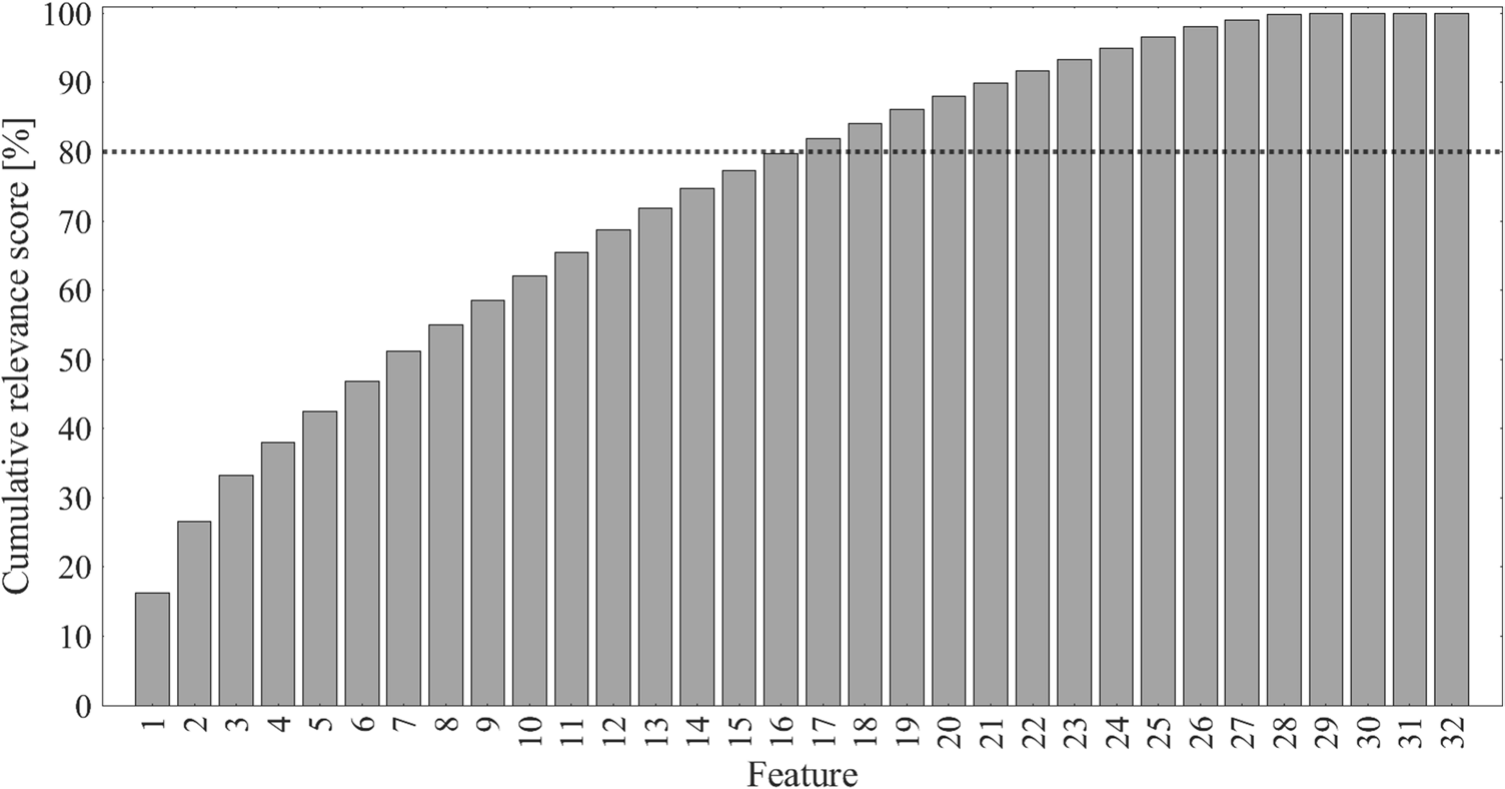
Cumulative relevance score (percentage) for all the extracted features, from the most relevant to the less relevant one. Specifically: (1) MNP, (2) mean A_pp_ in *C*_*P*_, (3) fragmentation, (4) standard deviation of the five highest CWT powers, (5) standard deviation of A_pp_ in *C*_*P*_, (6) number of neighbors with A_pp_ > 1.5 mV, (7) LAT, (8) relative PSD power in 180–200 Hz, (9) relative PSD power in 200–220 Hz, (10) relative PSD power in 100–120 Hz, (11) EGM A_pp_, (12) weighted mean A_pp_ in *C*_*P*_, (13) sum of the five highest CWT powers, (14) relative PSD power in 140–160 Hz, (15) relative PSD power in 220–240 Hz, (16) relative PSD power in 80–100 Hz, (17) number of neighbors with A_pp_ < 0.5 mV, (18) relative PSD power in 280–300 Hz, (19) relative PSD power in 40–60 Hz, (20) PKF, (21) relative PSD power in 160–180 Hz, (22) relative PSD power in 120–140 Hz, (23) CD, (24) relative PSD power in 260–280 Hz, (25) relative PSD power in 300–320 Hz, (26) relative PSD power in 240–260 Hz, (27) MNF, (28) number of neighbors with 0.5 mV < A_pp_ < 1.5 mV, (29) PSR, (30) CV, (31) relative PSD power in 60–80 Hz, (32) relative PSD power in 0–20 Hz. Each bar represents the relevance score due to the features up to the one shown in the x-axis (i.e., the second bar corresponds to the relevance contributions of features #1 and #2 with respect to the total amount of relevance, and so on), except for the case of feature #1, in which the cumulative relevance score coincides with the relevance score assumed by this feature.

Figure 3 illustrates the features selected using the mRMR-based method by reporting their cumulative relevance scores. In accordance with the proposed feature selection approach, all features up to #16 were retained, because they cumulatively reached the 80% of the total relevance (black dotted line).

Table 1 reports the results of the three classifier models in the 10-time 10-fold cross-validation, both with all the features or after the feature selection. The classification performances among the three classifiers were high, similar, and stable, thus highlighting the robustness of the proposed features regardless of the adopted classification model. However, the results remained stable even when only some features were considered. Furthermore, among the classifiers, the ENS model provided the highest accuracy, TPRs, TNRs, and F1-scores, and the lowest FPRs overall. The values of TPRs and TNRs slightly differed according to the chosen classifier. In particular, the ENS model presented similar and balanced performance in both metrics, which were approximately 93% when no feature selection was performed, whereas they were above 91% when features were selected. The same trend was observed in the SVM, but only when no feature selection was performed. Conversely, the KNN model exhibited an imbalanced performance with higher TPR than TNR, thus allowing for a better recognition of AVPs than physiological EGMs.

**Table 1:** Classification performance in the 10-time 10-fold cross-validation.

	ACC (%)	TPR (%)	TNR (%)	FPR (%)	F1-score
All features					
SVM	89.4 ± 1.4	89.3 ± 0.9	89.6 ± 2.7	10.4 ± 2.7	0.89 ± 0.01
KNN	88.3 ± 0.9	93.0 ± 0.6	83.6 ± 1.5	16.4 ± 1.5	0.89 ± 0.01
ENS	93.1 ± 0.3	93.1 ± 0.7	93.1 ± 0.4	6.9 ± 0.4	0.93 ± 0.00
Selected features					
SVM	87.5 ± 4.6	90.6 ± 1.8	85 ± 11	15 ± 11	0.88 ± 0.03
KNN	87.8 ± 0.7	91.5 ± 0.5	84.2 ± 1.5	15.8 ± 1.5	0.88 ± 0.01
ENS	91.6 ± 0.6	91.4 ± 0.7	91.9 ± 1.0	8.1 ± 1.0	0.92 ± 0.01

Results in the 10-time 10-fold cross-validation are reported as mean and standard deviation for all performance indexes and approaches, being computed across the 10 iterations cumulatively. Evaluations were performed by considering all features, i.e., without any feature selection, and including only those features selected by the mRMR algorithm.

Table 2 gives an overview of the results in a possible real application scenario by exploiting the leave-one-subject-out cross-validation. The results remained high and stable when all features or only the selected ones were considered, and the ENS provided the highest performance. However, the TPR and TNR values when no feature selection was performed indicate that an imbalance can be observed for the ENS model, which better identified physiological potentials than AVPs (TNR = 88.1% vs. TPR = 79.4%), while the other two models performed homogeneously. Nonetheless, this imbalance was reduced when only the most relevant features were retained (TNR = 85.3% vs. TPR = 82.5%).

**Table 2:** Classification performance in the leave-one-subject-out cross-validation.

	ACC (%)	TPR (%)	TNR (%)	FPR (%)	F1-score
All features					
SVM	74.7	75.6	74.2	25.8	0.70
KNN	80.2	80.1	80.2	19.8	0.76
ENS	84.7	79.4	88.1	11.9	0.80
Selected features					
SVM	74.7	79.0	71.9	28.1	0.71
KNN	81.6	82.4	81.1	18.9	0.78
ENS	84.2	82.5	85.3	14.7	0.80

Results in the leave-one-subject-out cross-validation are provided as a single cumulative value for each performance index and approach by considering all features, i.e., without any feature selection, and including only those features selected by the mRMR algorithm in the 10-time 10-fold case.

## Discussion

In this work, several features were presented and assessed to investigate their effectiveness in designing and training an automatic system that can support clinicians in AVP identification during post-ischemic VT substrate mapping procedures. All features were conceived according to the electrophysiological characteristics of AVPs and physiological potentials, and thus extracted from different domains.

The results show that the adoption of multi-domain features that aim to recognize AVPs is feasible and effective, allowing for reliable and accurate recognition in more than 93% of cases when 10-time 10-fold cross-validation is considered (i.e., 89.4%, 88.3%, and 93.1% for SVM, KNN, and ENS models, respectively; see Table 1), and a very high rate of correctly recognized AVPs (i.e., 89.3%, 93.0%, and 93.1% for SVM, KNN, and ENS models, respectively) with low false positive alarms (i.e., 10.4%, 16.4%, and 6.9% for the SVM, KNN, and ENS models, respectively), while guaranteeing stable and precise identification (see standard deviation values in Table 1). These findings were further confirmed when the number of involved features was reduced, thus highlighting that the adopted feature selection strategy could lead to comparable and accurate performance with a reduced computational load. Similar results were obtained when a more restricted number of features (i.e., the five most relevant ones) was considered, with performance differences < 3.5% (Supplementary Table S3), thus paving the way for a light integration of the proposed approach in off-the-shelf systems.

A comparison with the classification results reported in a previous study^49^, with respect to the adoption of the time and time scale domain features only, indicates that our new features improved the recognition accuracy by about 10% on average. Their use even outperformed the adoption of an artificial neural network trained and tested on the time series of each EGM (+ 10% accuracy), thus confirming their effectiveness for AVP recognition.

When the performance on the leave-one-subject-out cross-validation was considered, which has the greatest resemblance to a real application scenario, a slight deterioration in all performance indexes can be observed, as expected, with greater differences between the accuracy and TPR values (i.e., a decrease of 10.4% and 13.4%, on average, respectively, when no feature selection is performed, and 8.8% and 9.9% when feature selection is adopted, respectively). The use of patient-related validation introduced a strong imbalance not only in terms of numerosity of AVPs and physiological potentials annotated by the cardiologist for each patient, but also in terms of EGMs annotated for each EA mapping procedure, thus producing an uneven split of the dataset into the training and testing sets. Therefore, the use of the leave-one-subject-out scheme led to models with lower generalization capabilities when applied to observations belonging to patients never seen during training, thus yielding lower performance. The SVM model showed the most relevant reduction in performance with respect to the 10-time 10-fold case. Furthermore, the difference between the classification model performances increased, so that the accuracy difference between the SVM and ENS models changed from 3.7 to 10.0% when no feature selection was performed and from 4.1 to 9.5% when feature selection was implemented (see Table 1). The same trend can also be observed in TNR values.

The literature already reported studies on feature-based approaches developed to support clinicians in the identification of VT arrhythmogenic sites, and emerging strategies to target points for VT ablation. However, a performance comparison is complex as these works present findings based on different metrics and populations, and the rationale is different. As a matter of fact, some studies aimed at the identification of cut-off thresholds on features or criteria modelled on VT and non-VT patients as control group^33–35,37^, which is very different from distinguishing between AVPs and physiological EGMs in the same post-ischemic VT population. Furthermore, some important works aimed at detecting ablation areas^33,41^, fractionated signals^38^, VT sites of origin^39,40^, or VT isthmuses^36^, which is not strictly the same aim of the present study. Some other studies investigated alternative targeting strategies for ablation^43,45,47^, for the choice of the ablation targets in prospective clinical studies, and their efficacy was evaluated in terms of the clinical response of the patients after ablation. In contrast with the approach presented in our study, which aims at detecting AVPs to assist clinicians in determining arrhythmogenic sites during EA mapping, these investigations actually aimed to identify ablation target points, which is beyond the scope of our work.

A retrospective analysis of the location of the arrhythmogenic sites identified by the best-performing model in the leave-one-subject-out validation scheme (i.e., the ENS classifier), with respect to the voltage map and to the sites ablated during the clinical procedure, has been carried out. Remarkably, being our study performed retrospectively, ablation points were selected regardless of the proposed computer-aided system, according to different clinical strategies.

**Figure 4:**
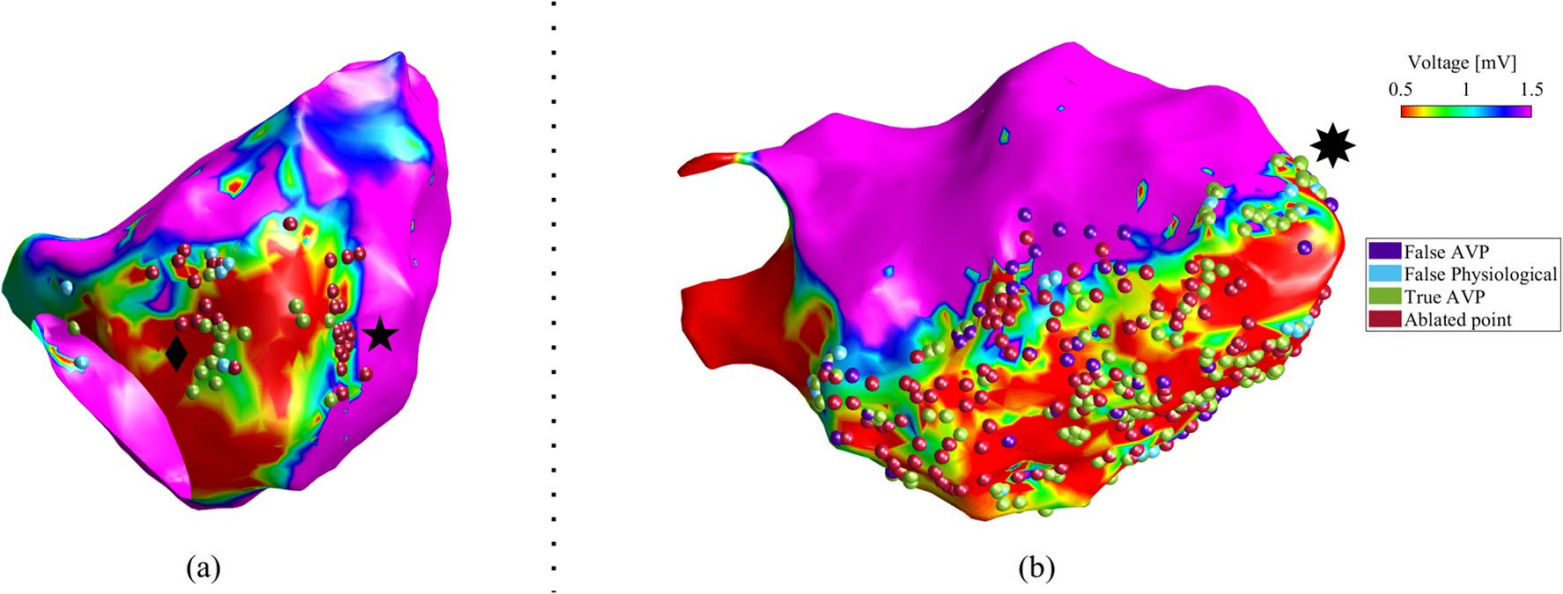
Some examples of voltage maps of procedures included in this work, in which AVPs correctly identified (green marbles), EGMs labelled as physiological but recognized as AVPs (purple marbles), EGMs annotated as AVPs but recognized as physiological (light blue marbles), and endocardial sites ablated during the procedure (red marbles) are depicted. On the left map (**a**), a channel entrance/exit to the slow conduction zone (black star) and a VT isthmus (black diamond) are depicted, while on the right map (**b**), a cluster of not-ablated AVPs located in apical LV area (black octagram) is highlighted. The EA voltage maps were generated from CARTO 3 export files in MATLAB v2022a (MathWorks Inc., MA, USA).

Indeed, the relevance of the proposed computer-aided identification of AVPs can be appreciated by looking at the examples reported in the Fig. 4. Specifically, in those cases where the scar-related substrate is not characterized by complex re-entry morphologies, as the one shown in Fig. 4a, the AVPs are quite well positioned onto the map. In that reported case, the clinician identified and ablated one channel entrance/exit to the slow conduction zone (black star in Fig. 4a) and a VT isthmus (black diamond in Fig. 4a). In this map, AVPs detected by the proposed system are coherently placed in correspondence of the entrance and in the inner portion of the isthmus. Consequentially, when the VT circuits are not complex, the value of the proposed system would be in speeding up the clinical procedure and supporting the easier identification of the re-entrant circuits in the scar. Conversely, an opposite situation can be observed in case of very complex VT circuits, as the one shown in Fig. 4b. In this case, lots of AVPs were identified by our system, predominantly near ablated regions. In contrast to this, during the ablation procedure, the AVPs located in apical LV area (black octagram in Fig. 4b) have been missed. Remarkably, in the same region, several AVPs have been correctly recognized by our tool, which would have been helpful to plan the subsequent ablation, if available in real-time. Looking more deeply at the same map, we can appreciate several EGMs annotated as physiological but recognized as AVPs by the ENS (purple marbles in Fig. 4b), mainly in deep scar areas. Here, even if the ENS output did not agree with the annotation, it is interesting to note that such false AVPs are surrounded by real AVPs and ablated sites, thus possibly reflecting some pathological aspects objectively identified by the model but not by the human operator, hence without overfitting the expert’s point of view. As such, by considering the typical low success rate of substrate ablation procedures during sinus rhythm (i.e., about 60–70%^18–20^), our study is encouraging since can support clinical expert in arrhythmogenic sites identification in complex VT circuits, where the human commitment is hampered, thus highlighting arrhythmogenic sites to be considered for optimal ablation strategy and outcome.

## Conclusion

This work demonstrated the feasibility of supervised machine learning tools for the identification of arrhythmogenic sites in post-ischemic VT patients, thus enabling the conception and adoption of computer-aided systems supporting clinicians and possibly improving the clinical outcome.

Different classification models for the automatic identification of AVPs in intracardiac bipolar EGMs were trained by using features from multiple domains. Classification performance revealed the robustness of the chosen features with respect to the different classification models, although some differences can be observed. High recognition capabilities are observable even when a leave-one-subject-out validation scheme is applied, which is closer to a real application scenario, albeit with a performance deterioration, particularly in terms of accuracy and TPR. Furthermore, arrhythmogenic sites identified by the best-performing model in the leave-one-subject-out validation scheme have been contextualized in the procedure in terms of location on the EA map and position of the real ablation targets, confirming the potentiality in assisting clinical experts during EA mapping. This study has two main limitations, namely, the dataset size and the single-expert annotation, which could have biased the results. As such, dataset enlargement is needed, including multiple annotators for the labeling process, to confirm the results of this study.

Despite being evaluated in a retrospective study, our findings are encouraging, suggesting the possibility of AVP automatic recognition with the proposed features, the conception of an AI-based computer-aided systems that support clinicians in VT interventional cardiology, and also possibly the improvement of clinical outcomes. On this regards, larger studies are needed to prospectively evaluate the clinical value of introducing the proposed machine learning tools in supporting clinicians in the identification of arrhythmogenic sites for VT ablation during electrophysiological procedures.

## Supplementary Information

Supplementary Tables.

## Data Availability

The datasets generated during and/or analyzed during the current study are available from the corresponding author on reasonable request.

## References

[1] Koplan BA, Stevenson WG (2009). Ventricular tachycardia and sudden cardiac death. Mayo Clin. Proc..

[2] Benjamin EJ (2019). Heart disease and stroke statistics—2019 update: A report from the American Heart Association. Circulation.

[3] Virani SS (2020). Heart disease and stroke statistics—2020 update: A report from the American Heart Association. Circulation.

[4] de Bakker JMT (1993). Slow conduction in the infarcted human heart. ‘Zigzag’ course of activation. Circulation.

[5] Harris P, Lysitsas D (2015). Ventricular arrhythmias and sudden cardiac death. BJA Educ..

[6] Wissner E, Stevenson WG, Kuck K-H (2012). Catheter ablation of ventricular tachycardia in ischaemic and non-ischaemic cardiomyopathy: Where are we today? A clinical review. Eur. Heart J..

[7] Briceño DF (2017). Substrate ablation of ventricular tachycardia: Late potentials, scar dechanneling, local abnormal ventricular activities, core isolation, and homogenization. Card. Electrophysiol. Clin..

[8] Guandalini GS, Liang JJ, Marchlinski FE (2019). Ventricular tachycardia ablation: Past, present, and future perspectives. JACC. Clin. Electrophysiol..

[9] Tung R (2015). Freedom from recurrent ventricular tachycardia after catheter ablation is associated with improved survival in patients with structural heart disease: An International VT Ablation Center Collaborative Group Study. Hear. Rhythm.

[10] Kuck K-H (2010). Catheter ablation of stable ventricular tachycardia before defibrillator implantation in patients with coronary heart disease (VTACH): A multicentre randomised controlled trial. Lancet (London, England).

[11] Martinez BK (2020). Systematic review and meta-analysis of catheter ablation of ventricular tachycardia in ischemic heart disease. Hear. Rhythm.

[12] Sapp JL (2016). Ventricular tachycardia ablation versus escalation of antiarrhythmic drugs. N. Engl. J. Med..

[13] Jaïs P (2012). Elimination of local abnormal ventricular activities: A new end point for substrate modification in patients with scar-related ventricular tachycardia. Circulation.

[14] Vergara P (2012). Late potentials abolition as an additional technique for reduction of arrhythmia recurrence in scar related ventricular tachycardia ablation. J. Cardiovasc. Electrophysiol..

[15] Cheung JW (2020). Targeting abnormal electrograms for substrate-based ablation of ventricular tachycardia: Can we ablate smarter?. Clin. Electrophysiol..

[16] Komatsu Y (2014). Substrate-based approach for ventricular tachycardia in structural heart disease: Tips for mapping and ablation. J. Arrhythm..

[17] Muser D, Santangeli P, Liang JJ (2017). Management of ventricular tachycardia storm in patients with structural heart disease. World J. Cardiol..

[18] Fernandez-Armenta J (2020). Safety and outcomes of ventricular tachycardia substrate ablation during sinus rhythm: A prospective multicenter registry. JACC. Clin. Electrophysiol..

[19] Haanschoten DM (2019). Long-term outcome of catheter ablation in post-infarction recurrent ventricular tachycardia. Scand. Cardiovasc. J..

[20] Radinovic A (2022). Matching ablation endpoints to long-term outcome: The prospective multicenter Italian ventricular tachycardia ablation registry. JACC. Clin. Electrophysiol..

[21] Shivkumar K (2019). Catheter ablation of ventricular arrhythmias. N. Engl. J. Med..

[22] Issa ZF, Miller JM, Zipes DP (2009). Clinical Arrhythmology and Electrophysiology. A Companion to Braunwald’s Heart Disease.

[23] Natale A, Wazni OM (2007). Handbook of Cardiac Electrophysiology.

[24] Aziz Z, Tung R (2018). Novel mapping strategies for ventricular tachycardia ablation. Curr. Treat. Options Cardiovasc. Med..

[25] Huang SKS, Miller JM (2019). Catheter Ablation of Cardiac Arrhythmias.

[26] Sacher F (2015). Substrate mapping and ablation for ventricular tachycardia: The LAVA approach. J. Cardiovasc. Electrophysiol..

[27] Tsiachris D (2015). Electroanatomical voltage and morphology characteristics in postinfarction patients undergoing ventricular tachycardia ablation: Pragmatic approach favoring late potentials abolition. Circ. Arrhythm. Electrophysiol..

[28] Haqqani HM (2009). Fundamental differences in electrophysiologic and electroanatomic substrate between ischemic cardiomyopathy patients with and without clinical ventricular tachycardia. J. Am. Coll. Cardiol..

[29] Luther V (2016). A prospective study of ripple mapping the post-infarct ventricular scar to guide substrate ablation for ventricular tachycardia. Circ. Arrhythm. Electrophysiol..

[30] Bourier F (2019). Is it feasible to offer ‘targeted ablation’ of ventricular tachycardia circuits with better understanding of isthmus anatomy and conduction characteristics?. EP Eur..

[31] Launer H, Clark T, Dewland T, Henrikson CA, Nazer B (2019). An automated fractionation mapping algorithm for mapping of scar-based ventricular tachycardia. Pacing Clin. Electrophysiol..

[32] Martin C (2019). Use of novel electrogram “Lumipoint” algorithm to detect critical isthmus and abnormal potentials for ablation in ventricular tachycardia. JACC Clin. Electrophysiol..

[33] Zeppenfeld K (2005). Identification of successful catheter ablation sites in patients with ventricular tachycardia based on electrogram characteristics during sinus rhythm. Hear. Rhythm.

[34] Lin C-Y (2016). Simultaneous amplitude frequency electrogram transformation (SAFE-T) mapping to identify ventricular tachycardia arrhythmogenic potentials in sinus rhythm. JACC Clin. Electrophysiol..

[35] Campos B, Jauregui ME, Marchlinski FE, Dixit S, Gerstenfeld EP (2015). Use of a novel fragmentation map to identify the substrate for ventricular tachycardia in postinfarction cardiomyopathy. Hear. Rhythm.

[36] Kuroki K (2018). New substrate-guided method of predicting slow conducting isthmuses of ventricular tachycardia: Preliminary analysis to the combined use of voltage limit adjustment and fast-fourier transform analysis. Circ. Arrhythm. Electrophysiol..

[37] Gupta, D., Hashemi, J., Akl, S. & Redfearn, D. A novel method for automated fractionation detection in ventricular tachycardia. in *2016 Computing in Cardiology (CinC)* 925–928 (IEEE, 2016).

[38] Gupta D (2016). Novel automated paced fractionation detection algorithm for ablating ventricular tachycardia. J. Biomed. Sci. Eng..

[39] Orini M (2020). Evaluation of the reentry vulnerability index to predict ventricular tachycardia circuits using high-density contact mapping. Hear. Rhythm.

[40] Campos FO (2019). Characterizing the clinical implementation of a novel activation-repolarization metric to identify targets for catheter ablation of ventricular tachycardias using computational models. Comput. Biol. Med..

[41] Masjedi M (2021). A novel algorithm for 3-D visualization of electrogram duration for substrate-mapping in patients with ischemic heart disease and ventricular tachycardia. PLoS ONE.

[42] Jackson N (2015). Decrement evoked potential mapping. Circ. Arrhythm. Electrophysiol..

[43] Porta-Sánchez A (2018). Multicenter study of ischemic ventricular tachycardia ablation with decrement-evoked potential (DEEP) mapping with extra stimulus. JACC. Clin. Electrophysiol..

[44] Bhaskaran A (2020). Decrement evoked potential mapping to guide ventricular tachycardia ablation: Elucidating the functional substrate. Arrhythm. Electrophysiol. Rev..

[45] Aziz Z (2019). Targeted ablation of ventricular tachycardia guided by wavefront discontinuities during sinus rhythm. Circulation.

[46] Acosta J (2018). Elucidation of hidden slow conduction by double ventricular extrastimuli: A method for further arrhythmic substrate identification in ventricular tachycardia ablation procedures. Eur. Eur. Pacing Arrhythm Card. Electrophysiol. J. Work. Groups Card. Pacing Arrhythm Card. Cell. Electrophysiol. Eur. Soc. Cardiol.

[47] Acosta J (2020). Long-term outcomes of ventricular tachycardia substrate ablation incorporating hidden slow conduction analysis. Hear. Rhythm.

[48] Baldazzi, G., Orrù, M., Matraxia, M., Viola, G. & Pani, D. Automatic recognition of ventricular abnormal potentials in intracardiac electrograms. in *2019 Computing in Cardiology (CinC)*, pp 1–4 (2019). 10.23919/CinC49843.2019.9005500.

[49] Baldazzi, G., Orrù, M., Matraxia, M., Viola, G. & Pani, D. Supervised classification of ventricular abnormal potentials in intracardiac electrograms. in *2020 Computing in Cardiology (CinC)*, pp 1–4 (2020). 10.22489/CinC.2020.397.

[50] Baldazzi G (2022). Spectral characterisation of ventricular intracardiac potentials in human post-ischaemic bipolar electrograms. Sci. Rep..

[51] Biosense Webster Inc. CARTO 3 System Instructions for Use, Software Version 6.0.80 UG-5400-006H (06A). https://www.e-ifu.com/shared-document/3656/2/0/shared?langcode=en&country=7511 (2021).

[52] Silberbauer J (2014). Noninducibility and late potential abolition: A novel combined prognostic procedural end point for catheter ablation of postinfarction ventricular tachycardia. Circ. Arrhythm. Electrophysiol..

[53] Marchlinski FE, Callans JD, Gottlieb CD, Erica Z (2000). Linear ablation lesions for control of unmappable ventricular tachycardia in patients with ischemic and nonischemic cardiomyopathy. Circulation.

[54] Roca-Luque I (2022). Accuracy of standard bipolar amplitude voltage thresholds to identify late potential channels in ventricular tachycardia ablation. J. Interv. Card. Electrophysiol..

[55] Stevenson WG (1989). Fractionated endocardial electrograms are associated with slow conduction in humans: Evidence from pace-mapping. J. Am. Coll. Cardiol..

[56] Gardner PI, Ursell PC, Fenoglio JJJ, Wit AL (1985). Electrophysiologic and anatomic basis for fractionated electrograms recorded from healed myocardial infarcts. Circulation.

[57] de Bakker JMT (1988). Reentry as a cause of ventricular tachycardia in patients with chronic ischemic heart disease: Electrophysiologic and anatomic correlation. Circulation.

[58] Anter E, Tschabrunn CM, Buxton AE, Josephson ME (2016). High-resolution mapping of postinfarction reentrant ventricular tachycardia. Circulation.

[59] Baldazzi, G., Orrù, M., Matraxia, M., Viola, G. & Pani, D. Efficacy of spectral signatures for the automatic classification of abnormal ventricular potentials in substrate-guided mapping procedures. in *2022 Computing in Cardiology (CinC)* 1–4 (2022). 10.22489/CinC.2022.351.

[60] Phinyomark, A., Thongpanja, S., Hu, H., Phukpattaranont, P. & Limsakul, C. The usefulness of mean and median frequencies in electromyography analysis. in *Computational Intelligence in Electromyography Analysis: A Perspective on Current Applications and Future Challenges*, pp 195–220 (2012). 10.5772/50639.

[61] Masé, M. & Ravelli, F. Automatic reconstruction of activation and velocity maps from electro-anatomic data by radial basis functions. in *2010 Annual International Conference of the IEEE Engineering in Medicine and Biology*, pp 2608–2611 (IEEE, 2010). 10.1109/IEMBS.2010.5626616 21096180

[62] Williams SE (2021). OpenEP: A cross-platform electroanatomic mapping data format and analysis platform for electrophysiology research. Front. Physiol..

[63] Cantwell CD (2015). Techniques for automated local activation time annotation and conduction velocity estimation in cardiac mapping. Comput. Biol. Med..

[64] Chandrashekar G, Sahin F (2014). A survey on feature selection methods. Comput. Electr. Eng..

[65] Radovic M, Ghalwash M, Filipovic N, Obradovic Z (2017). Minimum redundancy maximum relevance feature selection approach for temporal gene expression data. BMC Bioinform..

