# Supplementary material for: Computer-aided detection of arrhythmogenic sites in post-ischemic ventricular tachycardia
Source: Sci Rep. 2023 Apr 27;13:6906. doi: 10.1038/s41598-023-33866-w (PMC10140038; doi:10.1038/s41598-023-33866-w)
Supplement: Supplementary file 1 — Supplementary Tables. [file 41598_2023_33866_MOESM1_ESM.docx]

Supplementary Material of the article

“Computer-aided detection of arrhythmogenic sites in post-ischemic ventricular tachycardia”

Giulia Baldazzi^1,2,†*^, Marco Orrù^1,2,†^, Graziana Viola^3^, Danilo Pani^1^

^1^Medical Devices and Signal Processing (MeDSP) Lab, Department of Electrical and Electronic Engineering (DIEE), University of Cagliari, Cagliari, Italy

^2^Department of Informatics, Bioengineering, Robotics and Systems Engineering (DIBRIS), University of Genoa, Genoa, Italy

^3^Department of Cardiology, Santissima Annunziata Hospital, Sassari, Italy

^*^Correspondence:

Giulia Baldazzi, Ph.D.

giulia.baldazzi@unica.it

^†^These authors contributed equally to this work

**Supplementary Table S1.** Classification performance of the SVM model with different kernels.

Classification performance obtained by the SVM model with linear kernel, radial basis function (RBF) kernel, and with polynomial kernel of 2^nd^ and 3^rd^ order. For the 10-time 10-fold (10T10F) cross-validation, mean and standard error for all performance indexes and approaches are reported, being computed across the ten iterations cumulatively. Conversely, for the leave-one-subject-out (LoSo) cross-validation, a single cumulative value for each performance index and approach is provided. As can be seen, the 3^rd^-order polynomial kernel provided stable and high results in both cross-validations, and as such it has been chosen for the subsequent analysis.

|  |  | ACC [%] | TPR [%] | TNR [%] | FPR [%] | F1-score |
| --- | --- | --- | --- | --- | --- | --- |
| 10T10F | Linear | 87.0 ± 0.6 | 87.5 ± 0.6 | 86.5 ± 1.3 | 13.5 ± 1.3 | 0.87 ± 0.01 |
|  | RBF | 83.8 ± 0.7 | 98.5 ± 0.2 | 69.2 ± 1.3 | 30.8 ± 1.3 | 0.86 ± 0.01 |
|  | 2^nd^-order polynomial | 90.1 ± 0.8 | 89.8 ± 1.0 | 90.4 ± 1.4 | 9.6 ± 1.4 | 0.90 ± 0.01 |
|  | 3^rd^-order polynomial | 89.4 ± 1.4 | 89.3 ± 0.9 | 89.6 ± 2.7 | 10.4 ± 2.7 | 0.89 ± 0.01 |
| LoSo | Linear | 83.8 | 86.7 | 81.9 | 18.1 | 0.81 |
|  | RBF | 65.2 | 13.4 | 98.3 | 1.7 | 0.23 |
|  | 2^nd^-order polynomial | 72.0 | 74.1 | 70.6 | 29.4 | 0.67 |
|  | 3^rd^-order polynomial | 74.7 | 75.6 | 74.2 | 25.8 | 0.70 |

**Supplementary Table S2.** Classification performance of the KNN model with different numbers of neighbors.

Classification performance obtained by the KNN model with a number of neighbors (k) equal to 5, 10 and the square root of the numerosity of the samples constituting the training data (T). For the 10-time 10-fold (10T10F) cross-validation, mean and standard error for all performance indexes and approaches are reported, being computed across the ten iterations cumulatively. Conversely, for the leave-one-subject-out (LoSo) cross-validation, a single cumulative value for each performance index and approach is provided. As can be seen, a number of neighbors equal to 10 provided stable and high results in both cross-validations, and as such it has been chosen for the subsequent analysis.

|  |  | ACC [%] | TPR [%] | TNR [%] | FPR [%] | F1-score |
| --- | --- | --- | --- | --- | --- | --- |
| 10T10F | k = 5 | 90.3 ± 0.8 | 91.5 ± 0.8 | 89.1 ± 1.4 | 10.9 ± 1.4 | 0.90 ± 0.01 |
|  | k = 10 | 88.3 ± 0.9 | 93.0 ± 0.6 | 83.6 ± 1.5 | 16.4 ± 1.5 | 0.89 ± 0.01 |
|  | k = sqrt(T) | 86.4 ± 0.6 | 91.2 ± 0.5 | 81.5 ± 1.3 | 18.5 ± 1.3 | 0.87 ± 0.01 |
| LoSo | k = 5 | 78.8 | 77.5 | 79.6 | 20.4 | 0.74 |
|  | k = 10 | 80.2 | 80.1 | 80.2 | 19.8 | 0.76 |
|  | k = sqrt(T) | 81.4 | 79.9 | 82.3 | 17.7 | 0.77 |

**Supplementary Table S3.** Classification performance obtained in the 10-time 10-fold cross-validation by considering the five most relevant features.

Classification performance obtained by the SVM, KNN and ENS models in the 10-time 10-fold cross-validation by considering the five most relevant features. Mean and standard error for all performance indexes and approaches are reported, being computed across the ten iterations cumulatively.

|  | ACC [%] | TPR [%] | TNR [%] | FPR [%] | F1-score |
| --- | --- | --- | --- | --- | --- |
| SVM | 87.5 ± 0.7 | 88.9 ± 1.0 | 86.1 ± 1.5 | 13.9 ± 1.5 | 0.88 ± 0.01 |
| KNN | 87.8 ± 0.8 | 91.4 ± 0.7 | 84.2 ± 1.8 | 15.8 ± 1.8 | 0.88 ± 0.01 |
| ENS | 89.8 ± 0.9 | 90.0 ± 1.0 | 89.5 ± 1.0 | 10.5 ± 1.0 | 0.90 ± 0.01 |
